# COVID-19 on the Nile: Review on the Management and Outcomes of the COVID-19 Pandemic in the Arab Republic of Egypt from February to August 2020

**DOI:** 10.3390/ijerph18041588

**Published:** 2021-02-08

**Authors:** Yai-Ellen Gaye, Christopher Agbajogu, Reida El Oakley

**Affiliations:** 1 Institute of Global Health, University of Geneva, Campus Biotech, Chemin des Mines 9, 1202 Genève, Switzerland; 2 Medical Faculty, Libyan International Medical University, Benghazi BEN218, Libya; eloakley@icloud.com

**Keywords:** COVID-19, SARS-CoV-2, pandemc, Egypt

## Abstract

As the world fights the coronavirus disease 2019 (COVID-19) pandemic caused by severe acute respiratory syndrome coronavirus 2 (SARS-CoV-2), the World Health Organization (WHO) reports that over 17 million people globally were infected with SARS-CoV-2 as of 1 August 2020. Although infections are asymptomatic in 80% of cases, severe respiratory illness occurs in 20% of cases, requiring hospitalization and highly specialized intensive care. The WHO, under the International Health Regulations, declared this pandemic a public health emergency of international concern; it has affected nearly all health systems worldwide. The health system in Egypt, similar to many others, was severely challenged when confronted with the need for urgent and major expansion required to manage such a significant pandemic. This review uses publicly available data to provide an epidemiological summary of the COVID-19 pandemic behavior during the first wave of the outbreak in Egypt. The article covers mathematical modeling predictions, Egypt’s healthcare system, economic and social impacts of COVID-19, as well as national responses that were crucial to the initial containment of the pandemic. We observed how the government managed the outbreak by enhancing testing capacity, contact tracing, announcing public health and social measures (PHSMs), as well as allocating extra funds and human resources to contain SARS-COV-2. Prospectively, economic losses from major sources of revenues—tourism, travel, and trade—may be reflected in future timelines, as Egypt continues to control cases and loss of life from COVID-19. Overall, trends indicate that the spread of COVID-19 in Egypt was initially contained. Revalidation of prediction models and follow-up studies may reveal the aftermath of the pandemic and how well it was managed in Egypt.

## 1. Introduction

In late December 2019, the World Health Organization (WHO)’s China Country Office was notified about reports of pneumonia of an unknown cause in Wuhan, China [1]. The cause of this pneumonia was later discovered to be a novel virus—severe acute respiratory syndrome coronavirus 2 (SARS-CoV-2)—which causes a clinical illness known as coronavirus disease 2019 (COVID-19) [1]. Following the extensive spread of the disease in neighboring Asian countries and parts of Europe, COVID-19 was declared a pandemic by the World Health Organization (WHO) on 11 March 2020. Although most cases are asymptomatic, this virus, which shares similarities with SARS-associated coronavirus (SARS-CoV) and Middle East respiratory syndrome–related coronavirus (MERS-CoV), can cause symptoms in infected persons. These symptoms range from mild, such as fever, malaise, dry cough, and shortness of breath, to severe, such as respiratory failure and acute myocardial injury, which can require medical attention and emergency hospitalization [1]. 

COVID-19 has spread all over the world, including Europe, the Americas, Australia, and, most recently, Africa. Egypt confirmed its first case of COVID-19 on 14 February 2020. Prior to that, no African country had reported a confirmed case. Egypt, being the first African country to report a confirmed case, was expected by other countries on the continent to set a precedence in creating a timely response to the virus. This makes a review on Egypt’s adherence to the international recommendations and improvised management of the outbreak noteworthy. By 1 May 2020, the virus spread to over 45 African countries and 21 Eastern Mediterranean region [2]. Egypt, therefore, was the second most affected African country (after South Africa), reporting 94,078 cases and 4805 deaths as of 1 August 2020 [2], whereas a total of 17,396,943 cases and 675,060 deaths were reported globally by the WHO at the same time [2]. 

This review utilizes publicly available and up-to-date data from several sources: published articles, WHO reports, news reports, Egypt’s official newspaper (Al Ahram), and other news reports, to provide insight into the trends of the COVID-19 pandemic in Egypt. The purpose of this study is to analyze the socioeconomic impacts of the significant measures/management techniques and outcomes elicited by COVID-19 in Egypt. This will include the epidemiological situation of the country, the healthcare system (preparedness, ranking, etc.), mathematical modeling predictions, the observed and expected economic, social, and political impacts, and, finally, non-pharmaceutical interventions put in place to control the spread of the virus. 

## 2. Core Part

### 2.1. Case Presentation 

#### 2.1.1. Country Characteristics

The Arab Republic of Egypt is a lower-middle-income country in northern Africa, with an estimated population of approximately 100 million (2020), making it the third-most populous country in Africa [3]. Egypt has a very young population, and between 1950 and 2020, life expectancy at birth increased among males and females to 70 and 75 years, respectively. Cairo—Egypt’s capital and largest city—hosts over 7 million inhabitants, while more than 20 million people live in the Greater Cairo metropolitan area. In 2019, Egypt’s population density was estimated to be 100 people per km^2^ [3]. The population of residents living in urban and rural areas increased between 1955 and 2020, with more individuals living in rural areas than urban areas. This highlights the limitations in accessing (and affording) high-quality healthcare [3]. The climatic condition of Egypt is characterized by cool temperatures and occasional rain during winter months (December to March), and hot, dry temperatures of ~49 degrees Celsius in summer months [3]. 

#### 2.1.2. Epidemiology and Mathematical Modeling of COVID-19 in Egypt 

The index case of COVID-19 in Egypt was reported on 14 February 2020, from a German citizen. In the wake of the first case discovery, countries, such as the United States of America, also reported cases associated with travels from Egypt [4]. On 8 March 2020, 48 COVID-19 cases were recorded, and the nation recorded its first COVID-19 related death: a 60-year-old German tourist. Following this, the transmission pattern changed from imported cases, and local transmission was established by mid-March 2020. The epidemic progression worsened from April to August 2020, as cases and deaths increased exponentially. On 1 April 2020, 710 cases and 46 deaths were reported. By 1 May 2020, a total of 5537 cases, including 269 deaths, were reported [2]. Among these cases, 45 were reported from an Egyptian river cruise ship, MS A’Sara, which was heading towards Luxor—a major tourist destination [4]. By the first of June, July, and August 2020, the COVID-19 cumulative case counts were 24,985, 68,311, and 94,078 respectively, with 959, 2953, and 4805 deaths reported, respectively [2]. The spike in cases in the population also increased the number of health worker infections and deaths. By June, four months into the outbreak, more than 400 COVID-19 cases amongst health workers were reported, as well as 111 deaths. This placed Egypt in 7th place out of 10, in countries with the highest COVID-19 health worker deaths [5]. 

**Figure 1:**
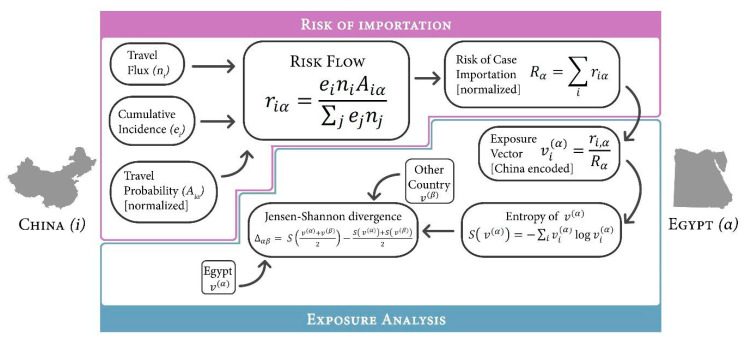
Adapted flow diagram of mathematical models used in determining the probability of exposure to imported coronavirus disease 2019 (COVID-19) from a city in China (*i*) to Egypt (*α*) [6].

**Figure 2:**
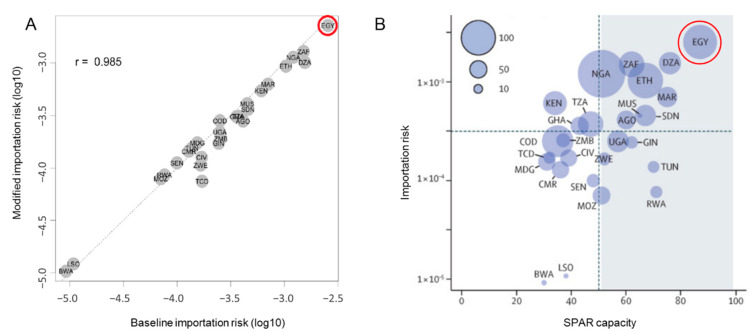
(**A**) Illustrates a scatter plot model of modified importation risk vs. baseline risk. Egypt is circled in red. (**B**) represents the importation risk of COVID-19 from China as a function of State Party Self-Assessment Annual Reporting (SPAR). Egypt is circled in red (reprinted with permission from Elsevier [6]. Copyright 2020 *The Lancet*).

Before the outbreak, a study was conducted to assess country vulnerability and preparedness against the importation of COVID-19. The WHO International Health Regulations (IHR) Monitoring and Evaluation Framework (MEF) was used to assess preparedness, whereas the Infectious Disease Vulnerability Index was representative of the country’s vulnerability. Using the mathematical model presented in Figure 1, the risk of COVID-19 importation into Egypt was measured as the probability of importing a case from an infected region of China (Figure 2A). Preliminary results showed Egypt as the country with the highest risk of case importation, with moderate capacity, alongside Algeria and South Africa (Figure 2B) [6]. 

To estimate the burden of COVID-19 in Egypt, a study was conducted at The University of Toronto’s Dalla Lana School of Public Health. The model used the exported case detection approach, involving 14 cases of COVID-19 in four countries: the USA, Taiwan, UAE, and France were linked to travels from Egypt [7]. Tuite and colleagues used data from the UN World Tourism Organization on the average length of stay of tourists in Egypt, and the proportion of travelers who were tourists, and not Egyptian residents. The model, which focused on a period between 6 February and 6 March 2020, estimated the burden of COVID-19 in Egypt to be as high as 19,310 (as the worst-case scenario), and 6000 cases (as the best-case scenario) [7]. The prediction of 6000 cases was almost consistent with reports of 5537 observed cases reported as of 1 May 2020 [2].

This was the first study conducted to predict COVID-19 burden in Egypt. However, the study was highly controversial and gave rise to public disagreement, prompting queries from researchers on some of the assumptions and methods used by Tuite and colleagues. Weaknesses in the study included the lack of “richness” of the data used, the difference in incubation periods, the unlikeliness of mixing between tourists and residents, and the strong possibility of individuals being infected before arrival in Egypt [7,8].

Samhoud reported that discrepancies in the model estimates could have been because the original model used 822 laboratory confirmed cases of the influenza A virus subtype H1N1, versus only three reported cases of COVID-19 [8]. He also added that, although H1N1 is a respiratory disease, like SARS-Cov-2, the former—which was used in the original model—has a shorter incubation period, and, therefore, could affect predictions.

Negida et al. touched on the controversy regarding mixing between tourists and residents, as touristic destinations are in “special” locations, characterized by small populations, and are typically far from densely populated residential areas [9]. He also mentioned that the data used from the United Nations World Tourism Organization (UNWTO) did not include the length of stay in February 2020. Hence, there is the strong possibility of these cases being individuals who were infected before arrival in Egypt—especially because airport screening for the virus was initially only applied to arrivals from China. The authors concluded by questioning the accuracy of the figures and the reliability of the conclusions made in the University of Toronto study [9].

Hassany et al., working at the Ministry of Health and Population, published a study with updated estimations of the COVID-19 burden in Egypt as of 31 March 2020, using different assumptions and measurements from Tuite and colleagues. Real-time fatality rates from 15 countries showed that, while USA and Germany employed “open-screening” to account for the fact that case fatality figures from Egypt could have been low due to the absence of open screening, 13 other Middle Eastern and North African countries shared close similarities to Egypt [10]. A factor (obtained by dividing the fatality rates of Egypt by the global fatality rate from the 15 countries mentioned) was multiplied by the current number of cases, arriving at an estimation of cases, ranging from 710 to 5241 as of 31 March 2020, indicating over-exaggeration of the original estimates. However, these figures could have been different due to the fact that the countries may have been at different stages of the epidemic, or used different management techniques, such as the number of PCR tests performed and related positivity rates, leading to different incidence and deaths rates over time [10].

#### 2.1.3. Healthcare System

Egypt’s pluralistic healthcare system is funded and managed by governmental, parastatal, and private sectors of the economy. The governmental and parastatal sectors are both run by the state and consist of hospitals, insurance organizations (Health Insurance Organization, HIO), and Curative Care Organization (CCO). The Health Insurance Organization (HIO) of the parastatal oversees basic health coverage for 60% of the population, for primary secondary and tertiary care [11].

In 2014, constitutional adjustments highlighted in the WHO health system profile of Egypt (2015) revitalized the health system, and improved the quality of care, health expenditure, availability, and accessibility of disease surveillance. This provides access to primary healthcare facilities that are within less than 5 km for 95% of the total population. Whereas total percentage expenditure on health to the Gross Domestic Product (GDP) remained the same, at 5.1% of GDP between 2005 and 2013 [12]. More recent data show that “Health expenditure per capita of Egypt increased from 54 US dollars in 2004 to 126 US dollars in 2018 growing at an average annual rate of 7.11%” [13].

Considering Egypt’s preparedness for outbreaks, useful indicators for assessment include disease prevention, detection, response, health system compliance, and vulnerability. A crude 2019 ranking with the mentioned indicators show Egypt possesses a Global Health Security (GHS) index score of 39.9 in pandemic preparedness. Therefore, it ranks 87th out of 195 countries globally, and 7th out of 54 African countries, according to the GHS Index [14]. In 2020, WHO assessed all countries on the preparedness and response status for COVID-19 based on two major real-time indicators: the country’s self-assessment and the continuous response to the risk of COVID-19 importation and transmission. In an assessment reported on 13 April 2020, Egypt was categorized—alongside Austria, Cyprus, France, Greece, Russia, and 22 other countries—at a preparedness capacity of level 4 out of 5 ≤80%, and a response category of 4 to tackle ≥10 cases [15]. In this response category, active cases in Egypt are linked with known sources of infection, with no report of community transmission of COVID-19 (not linked with either travel or close contact case exposure). This implies that, based on IHR national capacities, Egypt has a strong capacity to prevent new imported cases, as well as detect and respond to active cases to potentially mitigate the outbreak.

Regarding healthcare facilities and human resources, the United States Agency for International Development (USAID) in 2019, accounted for 86% of intensive care units in Egypt, participating in national surveillance of hospital-acquired infections and antimicrobial resistance. This makes up 435 intensive care units spread amongst 110 public hospitals and five private hospitals [16]. Moreover, in 2019, the public health system recorded a labor force of 72,900 physicians and 126,200 nurses [17]. Furthermore, a technical support mission conducted by the WHO in Egypt on 25 March 2020, for COVID-19 indicates that the country possesses strong disease surveillance and contact tracing systems to mitigate the spread of the disease. To ensure that the outbreak is controlled, the government allocated additional health professionals and extra funding for 21 testing laboratories to conduct 200,000 tests [18]. By June, the number of testing laboratories increased to 40. As part of the COVID-19 response, the Ministry of Health announced 340 hospitals nationwide were designated for treating COVID-19 cases, with plans to add more. Therefore, 35,152 beds, 2218 ventilators, and 3539 critical care beds were available for COVID-19 case management. By August, the number of public hospitals treating COVID-19 patients was 750 [5].

### 2.2. Management and Outcome

#### 2.2.1. Non-Pharmaceutical Intervention Measures Taken by Authorities.

Human exposure to coronavirus, as with many infectious diseases, needs to be avoided, especially in the absence of vaccines and antiviral agents. Strategies used by the Egyptian government include “flattening the curve” (managing and control the spread of the outbreak) through comprehensive and intensive contact tracing, rapid diagnosis, patient isolation, quarantine, social distancing, and other population-level strategies with the support of the community [19,20]. A summary of a timeline of key events that took place recently in Egypt, in effort to control the spread of the virus [18,19], is presented as follows:

- 26 January 2020: all flights from China to Egypt were banned.
- 19 March–31 March 2020: suspension of all flights in efforts to reduce tourism and disinfect major attractions in the country and hotels—the decision was made on 16 March 2020. Moreover, all public spaces (restaurants, cafes, casinos, nightclubs) were closed, and a 7 p.m.–6 a.m. curfew was enforced. All schools and universities were closed during the same period.
- 21 March 2020: official decision to close all places of worship (mosques and churches).
- 24 March 2020: The Ministry of Communications and Information Technology extended the air flight ban and closure of educational institutions for another two weeks (from the former 31 March deadline).
- 25 March 2020: A 7 p.m. to 6 a.m. curfew was enforced. This included all forms of transportation and specific services (e.g., including pharmacies, food points, and home delivery services).

Other continuous efforts included military personnel sterilizing public spaces and institutions in cities and encouraging public sector servants to work remotely, where possible.

The closure of all educational institutions, non-essential businesses, and religious houses, and the banning of large gatherings was put in place through the end of April 2020. However, the nighttime curfew, which was eventually shortened (8 p.m.–4 a.m.), remained in place until 27 June 2020.

Following this, the government introduced de-confinement measures and a phased plan to reopen the economy. In May, hotels, restaurants, and shopping activities resumed, with compliance to COVID-19 capacity, takeaway, and operating hours rules [19]. Government authorities tightened restrictions on the biggest religious celebrations of 2020 to prevent large gatherings and infections, during Eid Al-Fitr in May, and later in August for Eid Al-Adha. Whereas, between this period, Egypt’s airspaces reopened in July, accompanied by the reopening of leisure and entertainment centers (gyms, cinemas, etc.), whilst still enforcing strict maximum capacity limits (of 25%) and shortened operating hours. During this time, the government made mask-wearing mandatory in public spaces and transportation. Religious houses were also partially opened; public parks and beaches remained closed, but were scheduled to reopen at the end of August [19].

**Figure 3:**
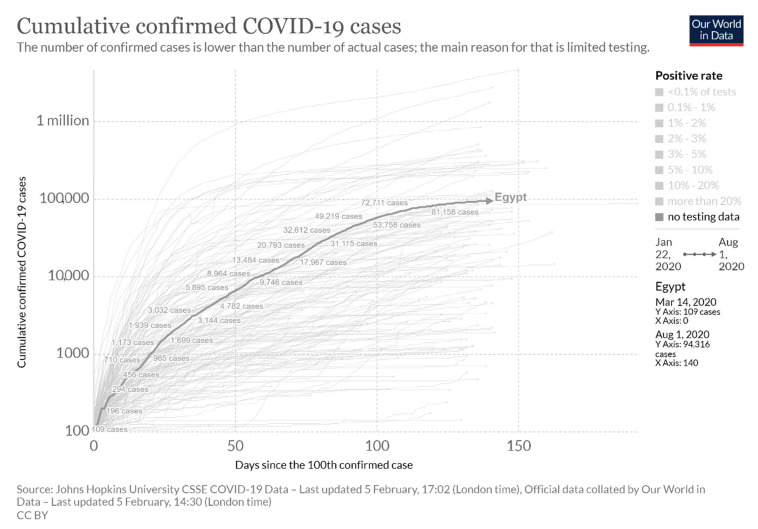
The trend of confirmed cases in Egypt, compared globally, between 22 January and 1 August 2020 (simulation by Routely, N., Virtual Capitalist: Healthcare [20]).

With the adoption of these measures, in addition to WHO guidelines concerning detection and quarantining of new cases, we can observe a simulation of the trajectory of Egypt in managing its epidemic and reducing the burden on its healthcare system (Figure 3) [20].

#### 2.2.2. Expected or Observed Impact on the Country’s Economy

This outbreak was expected to cause significant economic hardship worldwide, especially concerning international trade and travel, as many nations issued travel advisories—despite the WHO not approving any travel restrictions. According to Egyptian Prime Minister Mustafa Madbouly, the flight suspension was expected to incur about EGP 2.2 billion in losses.

Egypt’s lucrative tourism industry was making a turn-around after incessant political instability and flight bans, resulting from the 2011 Egyptian revolution and the 2015 bombing of a Russian airline over Egypt’s Sinai. A severe loss is expected to this significant part of Egypt’s revenue, worth EGP 174.1 billion (~$12.4 billion) in 2018, making up ~12% of the country’s GDP, and 10% of total employment [20,21]. At the beginning of the outbreak, the Egyptian government and central bank of Egypt announced stimulus policies of EGP 100 billion (~$6.4 billion) towards the anti-COVID-19 plan. Half of the stimulus funds, EGP 50 billion, was allocated to the tourism sector, as well as tax relief, to mitigate the damage [20,21]. EGP 3.8 billion ($241 million) was also granted to strengthen the healthcare sector—to equip hospitals with medical supplies to withstand shock, and to compensate medical staff, by increasing wages. In June 2020, an additional EGP 10 bn (~$636 million) in COVID-19 funds was added to the 2019/20 fiscal year budget. The majority of these funds were distributed amongst the ministries of education, transport, planning, and economic development, as well as the city of Cairo—which was one of the most affected regions in the country.

Other areas of the economy expected to face losses include revenue from the Suez Canal because of traffic disruption and remittances from Egyptian expatriates. Additional economic measures in place by the system include raising pensions by 14%, postponing payments; reducing interest rates on loans, housing, increasing payment limits, providing various options of debt and tax relief, and lowering energy costs for the industrial sector [20,21].

#### 2.2.3. Social and Political Disruption

Forecasting political consequences of COVID-19 poses a great challenge in such a dynamic and uncertain scenario; moreover, it was anticipated that disruptions during a global crisis would be magnified. News reports criticized the government’s lack of transparency regarding true figures and reporting fewer cases than what existed. This prompted suspicions from figures predicted in the University of Toronto study [7]. However, President Abdel Fattah el-Sisi reassured that the government was being entirely transparent, and all government statements reflected true figures. To avoid public panic, a statement was released by Egypt’s general prosecution stating that the spreading of fake news regarding COVID-19 could cost people up to EGP 20,000 in fines, in addition to possible imprisonment. Along with transparency concerns, more than seventy people were detained between March and June, including nine frontline responders who were critical to the COVID-19 response. Many of these arrests were made based on spreading “false news” through social media posts, in which detainees expressed their dissatisfaction with the government’s response or underlined their working conditions [22].

Social distancing rules also affect Egyptians, as social gatherings and celebrations are deeply embedded in the culture. This includes religious gatherings at mosques and churches, such as naming ceremonies, engagements, or communal prayers—especially on Fridays. In a study by the Pew Research Center, 62% of Egyptians reported attending worship services weekly, and 72% said religion plays a huge part in their lives [22]. To provide psychological support during this time, two hotlines have been allocated, thus far, by the Ministry of Health and Population.

Additionally, there is an inevitable increase in internet traffic as more people are connecting virtually to communicate with colleagues, friends, and family, who they would otherwise see in person. This includes schools and universities that are transitioning to virtual modes of teaching. The Ministry of Communications and Information Technology has offered free browsing on all educational platforms to make the transition easier and cheaper for all students.

## 3. Discussion

Globally, over 17 million confirmed cases of COVID-19 infections were reported to the World Health Organization (WHO) as of 1 August 2020. This has generated massive public fear and panic due to uncertainties and changes in the past months. Experts in political, economic, and social sciences attest to a new reality that global citizens will have to adapt to, regarding the coming recessions and losses. As a result, nation leaders are implementing policies to ensure that their countries can withstand this shock. Being the first African country to report a confirmed COVID-19 case in mid-February 2020, the government of Egypt closed airspaces, and implemented a partial-lockdown and curfew, putting a halt to many social gatherings. The measures implemented in early March may have contributed to the slow progression of the pandemic from February to April, followed by a sharp spike in cases, which was maintained throughout July. The rapid increase in cases coincided with the loosening of measures [19]. During the same period, health worker infections and deaths increased at an alarming rate.

As China was commended by the WHO on its transparency in the management and notification of its epidemic, the government of Egypt tried following suit with similar claims of total compliance and transparent reporting of official data. This was not received wholly due to restrictions on free journalism and measures taken by government officials to penalize the spreading of fake news, all stemming from suspicions raised by The University of Toronto study [7]. Although newer studies show lower estimates of potential infection rates [10], the data were still higher than the country’s official reports (approximately seven times). Skepticism and doubtfulness of the governmental response were heightened during March–June as lawyers, journalists, and health workers were arrested for criticizing the state’s response strategies and their poor working conditions. As health workers are essential to the response, an assessment to understand the possible causes of surging infections should be conducted. This will aid in the pandemic response, and in implementing better Infection Prevention and Control (IPC). Training for health workers on IPC for COVID-19 should be offered, and adequate stocks of personal protective equipment should be ensured.

Regardless of speculations, the government of Egypt and the citizens worked cohesively to control the outbreak by respecting social distancing measures [18,19]. We found no reports of non-compliance, such as protests outside places of worship (reported in other African countries). This is extremely important, as many measures require citizen cooperation. Compliancy towards social distancing rules, alongside the early halt on tourism—reducing the number of incoming foreigners and/or internal visitors—may have aided in the early control of the spread.

As governments struggle to lessen the economic losses and improve sanitary measures, they were tasked with managing the social impacts of COVID-19. For example, movement restrictions result in limited social gatherings and distancing from family and friends. This little-to-no human contact triggers loneliness amongst people who live alone, especially in elderly people and grandparents. Furthermore, the implementation of curfews, as well as the closure of businesses and public places (including places that encourage well-being on an individual level, such as restaurants, stores, parks, etc.), may have given rise to mental illnesses, such as depression, and may heighten anxiety in individuals who fear being infected. Although the measures have eased, the state should have interventions in place to address psychological needs amongst the population and frontline workers. Adapting to living with COVID-19 is a new reality people will face and adjustments may cause social damage—which may be harder to quantify than the economical damage.

The WHO recommendations on COVID-19 in the Eastern Mediterranean region prompted countries to test all suspected cases, take aggressive measures in tracing contacts, as well as ensure social isolation. However, considering that a majority of Egypt’s health system is over-run by private institutions, high costs for testing has become a burden for individuals who cannot afford to be tested. When it comes to the management of severe cases, when one observes the current state of Egypt’s healthcare system, it could have led to a worsening of the epidemic, especially in the absence of non-pharmaceutical measures. This is based on the GHS global ranking of Egypt’s health system on pandemic preparedness and a decline in the ratio of health professionals from 2009 to 2019. However, with combined guidelines from WHO, State Party Self-Assessment Annual Reporting (SPAR) assessments, and the absence of community transmission in the timeframe of this review, Egypt’s case-tracing and surveillance strategies show promising trends in the control of the outbreak [14,15,16,17,18]. Countries such as Italy and Spain, which were greatly affected, reported inefficiencies in surveillance and monitoring, which contributed to the increase in case fatality and unpreparedness in a healthcare capacity. Similarly, Egypt faced the same challenges despite increasing testing and case management centers. Therefore, human, technical, and infrastructure capacities may have been enhanced to meet the increasing demands and expectations from the health system. As commemorated in the global comparison (Figure 3), measures implemented early on in Egypt helped contain its rate of infection and minimize fatalities.

A limitation of this review includes limited access to real-time information and scientific articles on the COVID-19 pandemic in Egypt, as the first draft was authored in April 2020.

## 4. Conclusions

This review presents current trends of the on-going COVID-19 pandemic in Egypt (up until 1 May 2020). In a country of 100 million inhabitants, the government has taken necessary non-pharmaceutical measures to control spread, by limiting air traffic, closing schools and public spaces, and enforcing a nightly curfew. Despite rumors questioning the government’s transparency in reporting cases, they continue to assure citizens that official figures are a true depiction of the country’s reality. The government also announced relief packages to lessen the anticipated economic damage from COVID-19, and allocated half of the funds to its highly profitable tourism industry. In addition to the economic impact—arising from reduced tourism, distorted supply chains, remittances, health—there will also be societal changes—as a result of social-distances and movement restrictions. The pressure that COVID-19 will put on the (already fragile) healthcare system raises questions as to whether Egypt can deal with the outbreak. However, current trends, and a statement from the Minister of Population and Health, indicate that the spread can be contained. Overall, this highlights the significant measures taken by the nation to curb a potential increase in the rate of infection and sustain the economy. As this is a real-time review, predictions are subject to change as the situation progresses. The article only provides a snapshot of the COVID-19 situation during the first wave. Follow-up studies should explore how the outbreak progressed from August 2020, and how well the outbreak was managed and controlled in Egypt in comparison to other countries.
